# Salicaceae Endophytes Modulate Stomatal Behavior and Increase Water Use Efficiency in Rice

**DOI:** 10.3389/fpls.2018.00188

**Published:** 2018-03-02

**Authors:** Hyungmin Rho, Victor Van Epps, Nicholas Wegley, Sharon L. Doty, Soo-Hyung Kim

**Affiliations:** ^1^School of Environmental and Forest Sciences, College of the Environment, University of Washington, Seattle, WA, United States; ^2^Department of Biology, University of Washington, Seattle, WA, United States

**Keywords:** endophytes, rice, stomatal conductance, water potential, water relations, water use efficiency, water deficits, ABA

## Abstract

Bacterial and yeast endophytes isolated from the Salicaceae family have been shown to promote growth and alleviate stress in plants from different taxa. To determine the physiological pathways through which endophytes affect plant water relations, we investigated leaf water potential, whole-plant water use, and stomatal responses of rice plants to Salicaceae endophyte inoculation under CO_2_ enrichment and water deficit. Daytime stomatal conductance and stomatal density were lower in inoculated plants compared to controls. Leaf ABA concentrations increased with endophyte inoculation. As a result, transpirational water use decreased significantly with endophyte inoculation while biomass did not change or slightly increased. This response led to a significant increase in cumulative water use efficiency at harvest. Different endophyte strains produced the same results in host plant water relations and stomatal responses. These stomatal responses were also observed under elevated CO_2_ conditions, and the increase in water use efficiency was more pronounced under water deficit conditions. The effect on water use efficiency was positively correlated with daily light integrals across different experiments. Our results provide insights on the physiological mechanisms of plant-endophyte interactions involving plant water relations and stomatal functions.

## Introduction

Climate change has become a great challenge in agriculture by reducing potential yield of crops as environmental stresses on crops increase (Cai et al., 2015). Growing human population will reach 9.6 billion by 2050 (United Nations Department of Economic and Social Affairs, 2015), which ominously implies the demand for crop production will concurrently increase substantially. As a result, two central themes to food security must be addressed: (1) how to increase crop plant resiliency with dynamically changing environmental conditions, and (2) how to improve crop yield with more sustainable methods that possibly lessen the burden of chemical and irrigation inputs used for both fertilizers and environment management (Flexas, 2016).

Amongst all of the resources in the agricultural industry, field irrigation water usage draws the most attention of scientists and the public alike. Currently, 53% of cereal production is met by irrigation. If this trend continues, agriculture will remain as the biggest player in draining freshwater globally by 2050 (Rosegrant et al., 2009). Moreover, considering the fact that climate change brings unstable precipitation, more frequent runoffs, and weather extremes such as the 2012–2014 drought in California, United States (Griffin and Anchukaitis, 2014), it will necessitate more efficient, innovative approaches to water use.

Recent efforts in achieving higher crop sustainability involve increasing plant water use efficiency (WUE), including but not limited to, genetic manipulation of plants to form less stomata (Franks et al., 2015), selection of drought tolerant genotypes through fast screening methods (Condon et al., 2004), and alteration of canopy structure to maximize light acceptance (Drewry et al., 2014). Nevertheless, these techniques are not readily available in the field, still under testing, or maybe unrealistic for larger scale applications.

Endophytes are microorganisms living in the intercellular spaces of plants, providing several benefits to hosts, in turn, receiving carbohydrate-based nutrients for their growth (Dobereiner, 1992). These are mutualistic symbionts that can enhance plant fitness and performance in terms of increasing host biomass under stressful conditions (Goh et al., 2013). For example, endophytes that confer water stress tolerance are well studied (Rho et al., 2018) and a number of research articles show their efficacy in plant fitness and plasticity under low water availability (Khan A.L. et al., 2015; Khan et al., 2016; Gagné-Bourque et al., 2016). Thus, a realistic aim is to supplant other current agriculture methods with endophytic symbioses to increase crop WUE and yields.

Doty et al. (2009) isolated diazotrophic – di-nitrogen fixing – endophytic bacteria and yeast from the Salicaceae family of plants – native poplar (*Populus trichocarpa*) and willow (*Salix sitchensis*) growing in primary substrate of natural riparian zones. In this environment the nutrient supply to the plants was severely limited due to frequent flooding. The isolates were characterized in the paper and subsequent publications clearly demonstrated their potential symbiotic traits in other host species across taxa including grass species such as rice (Khan et al., 2012; Knoth et al., 2013, 2014). A major goal of our past and current studies has been to test the effectiveness of the endophytes from Salicaceae hosts on improving the growth and mitigating stress of host plants from other taxa, especially crops. Since then, we have been using these isolates to find out their potential benefits in other agricultural crops and to explore their mechanistic level impacts. In line with these efforts, our recent publication, Kandel et al. (2015), demonstrated that this endophyte consortium significantly increased biomass of the same rice variety, leading to the increases in yield potential. However, Kandel et al. (2015) did not provide information on the physiological benefits and their underlying mechanisms. In this context, the present study delivers a mechanistic view of having endophyte symbiosis focusing on water relations of rice as a host.

Previous results have provided evidence for prospective impacts of the select endophytes on plant functional traits. The select endophytes were shown to have multiple potentially symbiotic traits including phytohormone production (indole-3-acetic acid in Xin et al., 2009), biological nitrogen fixation (Knoth et al., 2014; Doty et al., 2016), and cross-host biological nitrogen fixation capacity (Knoth et al., 2013; Kandel et al., 2015). In a recent study (Khan et al., 2016), it was demonstrated *in vitro* with liquid chromatography that select endophytic bacterial and yeast strains produce plant hormones, including abscisic acid (ABA) – a key player in stomatal control and development. In the article, the authors inoculated poplar cuttings to test stomatal response and photochemical efficiency under water deficit conditions, examining daytime stomatal conductance (*g*_s_) and chlorophyll fluorescence (*F*_v_/*F*_m_). The results showed significant decreases in *g*_s_ over time and higher *F*_v_/*F*_m_ in inoculated cuttings compared to controls. Multiple possible mechanisms based on microbial assays and genomic analysis were provided. Based on the findings of endophytic ABA production, we postulate that these symbionts can trigger stomatal closure and affect stomatal development of host plants, as potential mechanisms for the observed drought tolerance.

Rice (*Oryza sativa*) is currently the second most important staple crop worldwide following maize (FAOSTAT, 2015). Since rice is suggested to be an isohydric species (Parent et al., 2010), it is likely to be sensitive to daytime water demand as increasing transpiration rate aligns with rising air temperature and light intensity – both leading to an increase in vapor pressure deficits (VPD) between the air and leaf surface – during the daytime. The control of losing and blocking water vapor exchange on the surface of leaves is important for managing the resource as well as operating photosynthetic machinery by absorbing enough amount of CO_2_ from the ambient air. Consequently, isohydric species have developed tighter governing of stomatal controls together with stable water potential adjustment to endure losing water during daytime, when gas exchanges of leaves occur most actively, and to survive in unfavorable water conditions (Tardieu and Davies, 1992).

Nonetheless, there are contradictory results about endophyte effects on stomatal control over numerous host plant species (Malinowski and Belesky, 2000). Some reported they facilitated the stomatal closure to conserve water inside, while others reported the opposite results; they assisted the hosts with opening stomata to absorb more CO_2_ from the atmosphere to photosynthesize and eventually leading to more biomass gain. However, since the experiments were conducted in different contexts with unquestionably various combinations between the hosts and symbionts, clear conclusions cannot be drawn.

To date, multiple studies examined the impacts of endophytes on whole plant physiology related to water relations and leaf gas exchange (Richardson et al., 1993; Elmi and West, 1995; Morse et al., 2002; Rogers et al., 2012), but their complete mechanisms matching with inoculated endophytes’ characteristics have not yet been demonstrated. To provide a comprehensive understanding of endophyte effects on host plant water relations, we conducted a series of greenhouse experiments to examine endophyte effects on physiological attributes of rice water relations: Stomatal responses, water potential, water use and biomass gain of rice plants upon endophyte inoculation. In this study, we tested hypotheses: (1) the ABA-producing endophytes reduce diurnal *g*_s_ leading to reduced total water use and improved WUE, (2) the inoculation effects on plant water relations differ between different endophyte strains, and (3) the effect size of endophyte inoculation varies with environmental conditions such as water deficits, elevated CO_2_, and light levels.

## Materials and Methods

Preparation of microbial materials for the four independent experiments shared the same protocols. All four experiments were conducted in a greenhouse in the Douglas Research Conservatory at the University of Washington (47°39′27″N, 122°17′21″W; 10 m elevation), Seattle, WA, United States. Details about the experimental settings are provided in **Table 1**.

**Table 1:** Experiment designs in this study.

Category	Experiment 1	Experiment 2	Experiment 3	Experiment 4
Growing season	Autumn to Spring (10/15/2014 – 3/28/2015)	Spring to Summer (3/21/2014 – 9/2/2014)	Summer to Autumn (7/14/2015 – 10/6/2015)	Spring to Summer (3/23/2017 – 7/20/2017)
Growing duration	158 days	165 days	84 days	119 days
Endophyte strains used	PTD1/WP5/WPB	WP5	*nifH* MIX (see **Table 2** for details)	*nifH* MIX (see **Table 2** for details)
Experimental settings	4 (CTRL/PTD1/WP5/WPB)	2 × 2 (CO2 × INOC)	2 × 2 (DRT × INOC)	2 (INOC)
Experimental environments	Greenhouse benches	Sunlit growing chambers	Greenhouse benches	Sunlit growing chambers
Nitrogen fertilization	1/4X N	1/4X N	1X N	1/4X N
Average RH (day/night)	48/54%	60/66%	57/71%	57/59%
Average air temperature (day/night)	22/19°C	23/19°C	29/20°C	21/17°C
Average instantaneous light intensity	174.0 μmol m^-2^ s^-1^	176.3 μmol m^-2^ s^-1^	313.7 μmol m^-2^ s^-1^	201.7 μmol m^-2^ s^-1^
Average daily light integral	9.5 mol m^-2^ d^-1^	9.1 mol m^-2^ d^-1^	35.8 mol m^-2^ d^-1^	10.8 mol m^-2^ d^-1^

### Origins of Endophytes and Preparation for Inoculation

Nine different strains of diazotrophic endophytic bacteria and yeast were used in this study whose *in vitro* characteristics were identified and reported previously by Doty et al. (2009). WP1, WP5, WP9, WP19, WPB, WW5, WW6, and WW7C were isolated from wild black cottonwood (*P. trichocarpa*) and wild willow trees (*S. sitchensis*) at their native habitat, the Snoqualmie River, Western Washington, whereas PTD1 was isolated from hybrid poplar (*P. trichocarpa* × *P. deltoides)* (Doty et al., 2005). Details about the endophyte strains are provided in **Table 2**. All of these endophyte strains have been shown to be potentially diazotrophic by having positive amplification of *nifH* marker gene for nitrogenase reductase (Doty et al., 2009; Knoth et al., 2013). Their positive effects on biomass increase of different host plants were also demonstrated by several articles in consequent studies (Khan et al., 2012, 2016; Knoth et al., 2014; Kandel et al., 2015; Khan Z. et al., 2015). In addition, their colonization efficiency on host plants was established and reported in our previous work (Knoth et al., 2013; Kandel et al., 2015, 2017). We used the same inoculation method that was developed from the prior studies.

**Table 2:** List of the endophyte strains used in this study.

Endophyte	Closest rRNA Match	Source	Reference	Experiment
PTD1	*Rhizobium* sp.	Hybrid poplar (*Populus trichocarpa* × *P. deltoids)*	Doty et al., 2005	1, 3, and 4
WPB	*Burkholderia* sp.	Wild poplar (*P. trichocarpa*)	Doty et al., 2009	1, 3, and 4
WP1	*Rhodotorula* sp.	Wild poplar (*P. trichocarpa*)	Khan et al., 2012	3 and 4
WP5	*Rahnella* sp.	Wild poplar (*P. trichocarpa*)	Doty et al., 2009	1, 2, 3, and 4
WP9	*Burkholderia* sp.	Wild poplar (*P. trichocarpa*)	Doty et al., 2009	3 and 4
WP19	*Acinetobacter* sp.	Wild poplar (*P. trichocarpa*)	Doty et al., 2009	3 and 4
WW5	*Sphingomonas* sp.	Wild willow (*Sitka sitchensis*)	Doty et al., 2009	3 and 4
WW6	*Pseudomonas* sp.	Wild willow (*S. sitchensis*)	Doty et al., 2009	3 and 4
WW7C	*Curtobacterium* sp.	Wild willow (*S. sitchensis*)	Doty et al., 2009	3 and 4

*The rRNA gene of each strain was compared with the BLAST NCBI database and identified as the closest match (Doty et al., 2009).*

The selected endophytes were grown on N-limited combined carbon medium (NL-CCM, Rennie, 1981) for growth of endophytes to maintain their nitrogen fixation ability. All of them grew well on the media, visually identified after 48-h growth, and then cell suspension cultures were started in flasks containing NL-CCM broth. Three to five days later, optical density of the bacterial culture was measured using a spectrophotometer (UV-1700, Shimazu America Inc., Columbia, MD, United States). The final concentration of the bacterial solution for inoculation was adjusted to OD_600_ = 0.1 (equivalent to approximately 1 × 10^7^ cells) using sterile deionized water and N-free liquid media (Doty et al., 2009). A mock-inoculum for the control group was prepared just with the N-free liquid media. All microbiological tasks were done in a sterile condition using proper aseptic sterilization techniques.

### Experiment 1: A Greenhouse Study Using Multiple Endophyte Strains

#### Preparation of Plant and Microbial Materials

Three bacteria strains were used in this greenhouse study. WP5, WPB, and PTD1 were selected to compare their effects on water relations of the host plants with emphasis on stomatal behaviors and leaf water potential components. Experiment 1 was conducted from October 15th 2014 to March 28th 2015.

We used a very early to early maturing, semi-dwarf, Japonica variety M-206 rice (*O. sativa*) that was identified as the best responding rice variety to the endophyte inoculation in a previous study (Kandel et al., 2015). Rice seeds were surface-sterilized by imbibing them with 3% NaOCl for 4 h to remove any debris and contaminants on the seed coat. The seeds were rinsed with sterilized deionized water for four times to wash out remaining NaOCl. Although this surface sterilization technique does not guarantee the removal of all microbes in the seeds due to possible internal/inherent microbiome inside (Nelson, 2017), it is a widely used procedure to eliminate at least the ones on the surface. Thus, any differences in responses from the plants can be statistically interpreted as the effects of endophyte inoculation treatments.

The surface sterilized seeds were planted in 1-gal pots placed into plastic buckets. Horticultural root media (Sunshine Mix #2, Sun Gro Horticulture, Agawam, MA, United States) were used to grow the plants. There were four treatments groups, three with single strain inoculations (WP5/WPB/PTD1) and a mock-inoculated control (CTRL) with ten replicates in each treatment group. As such, the total number of pots was forty, and eight plants were planted in a pot. The subjects were placed in a greenhouse bench space where the air temperature and relative humidity (RH) are automatically controlled. To account for the potential environmental gradient along a greenhouse bench, we used a randomized complete block design with blocks placed across the bench. To further minimize location effects of the greenhouse, the position of pots within a block was reset every week. The average air temperature of the greenhouse environment during the experiment was 22/19°C, 16/8 h day/night supplemented with high pressure sodium light (400 W single phase bulbs, Phillips Electronics North America Corp., Andover, MA, United States) to compensate for the lack of sufficient sunlight during the winter-time. The average daily light integral (DLI) was 9.5 mol m^-2^ d^-1^ of photosynthetically active radiation (PAR). The air temperature and light intensity were recorded in pendant type data loggers (UA-002-08, Onset Computer Corporation, Bourne, MA, United States) at 30-min intervals during the experiment period. Relative humidity was 48/54% day/night in average (**Table 1**).

After inoculation, plants were given 200 mL of a N-free liquid nutrient solution adjusted at the quarter-strength modified Hoagland solution (Hoagland and Arnon, 1950). Deionized water was fully given until the water filled up to a 10-cm mark of the buckets to simulate the field growing conditions of paddy soil growing rice. The amount of water and fertilizer supplied was recorded weekly.

#### Gas Exchange Measurements

We refer to Moldenhauer et al. (2013)’s classification to specify the growth and development stages of rice plants hereafter.

Gas exchange measurements were taken three times during the experiment on day 58 (V3-4), 118 (V5), and 153 (R1-2) using portable photosynthesis systems equipped with IRGAs (LI6400XT, Li-Cor, Inc., Lincoln, NE, United States). The measurements were taken between 12 pm and 5 pm each time. 2-cm^2^ leaf chamber fluorometers (6400-40, Li-Cor, Inc.) were set to measure photosynthetic assimilation rate (*A*), *g*_s_, and real-time intrinsic WUE (*A*/*g*_s_). Settings of the sensor heads were 1500 μmol m^-2^ s^-1^ PFD for saturating light intensity, 400 ppm of CO_2_ concentration in the reference cell of the instruments, 25°C block temperature, 300 μmol s^-1^ flow rate, and 40–70% RH to optimize the microclimate for photosynthesis during the measurements. Before the measurements, a minimum of 3 min was given for the response time of the leaf samples.

#### Stomatal Conductance Measurements

Hundred and sixty-three days after germination, at about R3-4 stage of their growth, the diurnal changes of *g*_s_ were measured at 3-h intervals from 9 am to 6 pm using steady state leaf porometers (SC-1, Decagon Devices, Inc., Pullman, WA, United States). The instruments were calibrated on site before the first measurements taken at 9 am.

#### Leaf Water Potential Measurements

One plant per pot was randomly selected to measure destructive leaf water potential to analyze water relations of the hosts that may be influenced by the microorganisms. A pressure bomb (Model-1000, PMS, Albany, OR, United States) was used to measure midday (12 pm to 1 pm) leaf water potential of the samples. The second or third youngest leaf from the top was chosen and immediately after the readings, the rest of the plants were harvested for taking measurements of solute potential of plant extracts. This approach was used to parse out osmotic potentials for the entire leaf, assuming homogenized whole plant solute potential is the same as the leaf solute potential. The harvested leaves were transported to a -80°C deep freezer for breaking the cell walls to mix apoplastic and symplastic solutions. The frozen samples were loaded and sapped in a hydraulic plant sap press (Plant Sap Press #2720, Spectrum Technologies, Inc., Aurora, IL, United States). Four hundred microliter of the extracted plant solution was collected in sample cups of a thermocouple psychrometer (SC-10, Decagon Devices, Inc.) to measure osmotic potential of the solution. Soluble sugar content (SSC) of the remainders was assessed by a handheld refractometer (RHB-10/ATC, Horiba, Japan).

#### Calculation of Stomatal Density

Specimens for evaluating influences of the bacteria on stomatal development of the hosts were collected using a common stomatal imprint technique (Gay and Hurd, 1975). The sampling was done 2 days after *g*_s_ was measured on day 165. The imprints were collected from the abaxial side of the leaves using nail polish. We counted stomata to calculate stomata density of the specimen. Three field of views were observed and the variables were counted for each sample. The triplicated observed variables were averaged to calculate the parameters. ImageJ program (Schneider et al., 2012) was used with an add-on package to count the numbers of stomata and epidermal cells of the specimen with 40X magnification from a standard compound microscope.

#### Verification of Colonization

General colonization characteristics of the endophytes and efficacy of the inoculation method used in the study are fully documented in Kandel (2016). Also, an extended review can be found in Kandel et al. (2017).

One rice plant per pot was harvested 99 days after germination to verify colonization of the endophytes. Leaf, stem, and root tissues were separately harvested and the surfaces of the tissues were sterilized by submerging them in 3% NaOCl for 3 min for leaf and stem tissues and 8 min for root tissues, followed by rinsing with sterilized deionized water four times to remove any remnants of NaOCl. The final rinsing water from each sample was collected to confirm the efficacy of the surface sterilization process. Approximately 100 mg of the sterilized tissues were transferred into 1.5-mL microtubes containing 400 μL NL-CCM solution and then they were ground with sterilized microtube pestles. The extracts were diluted into 10^-4^ using an aseptic serial dilution. The diluted solution was plated onto NL-CCM containing petri-dishes. After 48 h of incubation at room temperature, photos of the plates were taken on a photo stand. The photos were downloaded to further process colony forming unit (CFU) count. CFUs were counted with ImageJ program to compare the bacterial counts in the colonized tissues among the treatment groups. Total CFU count from the leaf, stem, root tissues combined was marginally higher for the inoculated plants compared to the control plants (*P* = 0.085).

### Experiment 2: A CO_2_ Enrichment Study Using a Single Endophyte Strain

A CO_2_ enrichment study was designed and conducted to test stomatal responses affected by endophytes under two different atmospheric CO_2_ concentration.

#### Preparation of Plant Materials

Experiment 2 was conducted from March 21st 2014 to September 2nd 2014. For each pot, surface sterilized four M-206 rice seeds were sown in a 3-gal plastic pot containing the same horticultural root media (Sunshine Mix #2, Sun Gro Horticulture) that was fully irrigated with tap water after seeding. Before placement in closed top chambers, the pots were nested in 4-gal plastic pails for easier measurements of fertilizer and water to be supplied. The watering and fertilizing were done through the gaps between the pots and the pails using a plastic funnel. The amount of water and fertilizer supplied was recorded weekly. A total of 32 pots were prepared; eight pots per chamber randomly assigned to receive control (E-) or endophyte (E+) treatments.

#### CO_2_ Treatment and Inoculation

The experiment was a 2 × 2 factorial with eight replications; two levels of atmospheric CO_2_ concentration – ambient CO_2_ (AMB, approximately targeted to 400 ppm) and elevated CO_2_ (ELE, app. 800 ppm) – and two levels of inoculation status – mock-inoculated control with surface sterilized seeds (E-) and diazotroph endophyte-inoculated treatment group (E+). Due to the nature of the CO_2_ treatment with a chamber, a split plot design was applied to deploy the pots in two sets of chambers. For the detailed environmental controls and specifications of the chambers, refer to Kinmonth-Schultz and Kim (2011) and Nackley et al. (2016).

A total of four chambers were used in the experiment. Two were AMB chambers with ambient air supplied through air ducts from outside of the greenhouse and the other two were set to ELE chambers with pure CO_2_ supplied through air tubing, of which concentration was controlled by flowmeters. Accordingly, a single chamber accommodated both four E- and four E+ plant pots; eight pots, plus a pot without plant samples for monitoring the amount of weekly evaporation from the soil surface by the airflow of the chambers.

The air temperature, RH, light intensity, and CO_2_ concentration were monitored and recorded in a data logger (CR1000, Campbell Scientific, Logan, UT, United States). The average temperature during the experiment was 23/19°C and RH of the air in the chambers was 60/66% day/night. The light regime of the greenhouse was set to 16/8 h day/night (7 am – 9 pm as a photoperiod) and the average DLI from the sunshine and the supplementary lighting of the facility was 9.1 mol m^-2^ d^-1^ of PAR. The average atmospheric CO_2_ concentrations of the two AMB chambers and the other two ELE chambers were 437/886 ppm, respectively. See **Table 1** for more details about the environmental settings.

In this experiment, one reference strain, WP5, was used. Seven days after sowing when 95% of the seeds were germinated, 2 mL of the prepared WP5 inoculum were added to each seedling. We pipetted the inoculum to the crown of the rice seedlings for the roots to easily absorb the solution. The other half of the plants were mock-inoculated with the same volume of the mock-inoculum for setting the control group.

As in Experiment 1, 200 mL of a quarter strength nitrogen with the nitrogen free medium was supplied and the plants were fully irrigated through the plastic pail to prevent any stress responses from water deficit.

#### Stomatal Conductance Measurements

At 128 days after germination around R3-4 stage of their growth, *g*_s_ of the youngest fully expanded leaves was measured at 3-h intervals to examine diurnal changes of the parameter using steady state leaf porometers (SC-1, Decagon Devices, Inc.). We applied the same measurement procedure described in Experiment 1.

### Experiment 3: A Water Deficit Study Using Endophyte Consortia

The third experiment was designed to test the endophyte effects on long-term WUE under both well-watered and water deficit conditions. The trial was conducted from July 14th to October 6th in 2015. The same plant material, M-206 rice, was prepared and used in this trial. The differences from the previous experiments were the endophyte treatment and the fertilization and irrigation conditions outlined below. Measured metrics were weekly pot-based transpiration and biomass allocation at harvest.

#### Preparation of Plant Materials and Inoculation/Water Deficit Treatments

The average air temperature recorded in data loggers (UA-002-08, Onset Computer Corporation) was 29/20°C in day/night. The average DLI was 35.8 mol m^-2^ d^-1^ of PAR with a 16/8 h day/night photoperiod of supplemental lighting. The air temperature and light intensity were recorded every 15 min. RH was 57/71% day/night in average (**Table 1**).

After the seed surface sterilization, four rice seeds were placed into 1-gal pots containing horticultural root media (Sunshine Mix #4, Sun Gro Horticulture). A total of 32 pots were prepared and placed into the same number of plastic buckets. Seven days after the germination, we inoculated the half of the samples with the prepared consortium of endophytes (**Table 2**). Targeted OD_600_ per strain was approximately 0.011, and so OD_600_ of the consortium of the selected nine strains was 0.1. Each plant received 2-mL of the consortium inoculum (MIX) using the inoculation technique outlined above. The other half was given the same volume of the mock-inoculum consisted of a N-free solution (CTRL).

Like Experiment 1, a randomized complete block design was applied to assign the pots on a bench in the greenhouse. Six pots without plants were located in the middle of the experimental design to collect weekly evaporation rate of the soil, and then the rate was used to calculate weekly transpiration during the experimental period. The total volumes of the water in the pots with plants were subtracted by the averaged volume of the water in the pots without plants every week to calculate weekly transpiration. Every week, 200 mL of the aforementioned fertilizer adjusted at a full strength nitrogen level of Hoagland was supplied and the pots were fully irrigated to 15-cm marks on the side of the buckets.

Six weeks after the germination, half of the pots were subjected to water deficit conditions. We stopped the weekly irrigation for these half (water deficit stressed, S), whereas continued full irrigation for the others (non-stressed, NS). From this point on, there were four treatment groups in the design: non-stressed control (NS_CTRL), non-stressed inoculated (NS_MIX), stressed control (S_CTRL), and stressed inoculated (S_MIX) with eight replications per each. To monitor the soil water status after the water deficit treatment, the soil water potential was measured by using a thermocouple psychrometer (SC-10, Decagon Devices, Inc.).

#### Water Use Efficiency Calculation

Weekly transpiration was recorded throughout the experimental period until the plants harvested after 4 weeks of induced water deficits. Total transpiration was calculated by adding up the weekly transpiration at harvest. Measured dry weights were divided by total transpiration after harvesting and 72-h drying at 70°C. The pot-based total transpiration and the measured dry weights were used to calculate long-term WUE.

$$\begin{array}{c} \;\mathrm{WUE}\;\mathrm{of}\;\mathrm{productivity}\;=\;\mathrm{total}\;\mathrm{biomass}\;\mathrm{gain}\;(g)/total\;\mathrm{transpiration}\;(L/pot) \end{array}$$

### Experiment 4: A Growth Chamber Study Using Endophyte Consortia

To provide support for a mechanistic understanding of the stomatal conductance responses, we repeated Experiment 2 in the previously used growth chamber of the same greenhouse facility from March 23rd 2017 to July 20th 2017. The objective of this experiment was to test the hypothesis that ABA produced endophyte consortia would increase *in vivo* ABA concentrations of the host plants.

#### Preparation of Plant Materials and Inoculation

For preparing the plant samples, the protocols from Experiment 2 were used. For preparing the microbial samples and inoculation process, the protocols from Experiment 3 were used. In short, a total of 32 rice plants were grown in 3-gal pots in four sunlit growing chambers. The half of the surface sterilized plants were inoculated by the *nifH* endophyte consortium 7 days after germination. They were grown under well-watered and nitrogen limited conditions until measurements and sampling taken over 104 days. The average air temperature recorded in a data logger (CR1000, Campbell Scientific) was 21/17°C in day/night. The average DLI was 10.8 mol m^-2^ d^-1^ of PAR with a 16/8 h day/night photoperiod of supplemental lighting. The air temperature and light intensity were recorded every 15 min. RH was 57/59% day/night in average. Further experimental details can be found in **Table 1**.

#### Stomatal Conductance Measurements

At 66 days after germination around V6-7 stage of their growth, *g*_s_ of the youngest fully expanded leaves was measured at 12 and 6 pm to examine diurnal changes of the parameter using steady state leaf porometers (SC-1, Decagon Devices, Inc.). We applied the same measurement procedure described in Experiments 1 and 2.

#### *In Vivo* ABA Assay

Diurnal *in vivo* ABA content was determined biochemically using the Phytodetek enzyme-linked immunosorbent assay (ELISA) kit (PDK 09347/0096, Agdia, Elkhardt, IN, United States). At 96 days after germination around R3-4 stage, the rice leaf samples were harvested at 12 pm and 6 pm. The fully expanded youngest leaves were immediately submerged into liquid nitrogen in centrifuge tubes. They were frozen and stored at -80°C until further analysis. The samples were ground into fine powder and approximately 100 mg of the powder of each was transferred into a microtube. ABA was extracted using 1 mL of 80% methanol at 4°C overnight. On the following day, the mixture was centrifuged at 10,000 rpm for 5 min. The supernatant was collected in a new microtube. The pellet was resuspended and 1 mL of fresh 80% methanol was used to repeat the extraction process at 4°C overnight. Again, by centrifuging the mixture at 10,000 rpm for 5 min and the supernatant was combined with the extracts from the previous day. The pooled supernatant was dried down using a vacuum concentrator until approximately 50 μL of liquid remained. Then, TBS buffer (25 mM Tris-HCl pH 7.5, 100 mM NaCl, 1 mM MgCl_2_, 3 mM NaN_3_) was added up to a final volume of 500 μL of the extract. The buffered extract was then diluted 10-fold in TBS buffer. The diluted sample was used to further detect ABA, following the Phytodetek ELISA assay kit manual. The ABA concentrations were measured using a multichannel spectrophotometer (Multiskan FC, Thermo Fisher Scientific, Waltham, MA, United States). Each sample (CTRL/MIX) at each time point was replicated eight times.

### Statistical Analysis

All physiology parameters measured in Experiments 1 through 4 were analyzed with R version 3.2.2 (R Development Core Team, 2015). For Experiment 1, the measures were subjected to contrast matrix to compare control vs. three single strain inoculated plants with blocking effect included in the model. For Experiment 2, the variables recorded were tested for the effects of a split-plotted CO_2_ treatment with a proper statistical analysis. The chamber effect on variation was found not to be significant *α* = 0.05 level. Consequently, two-way ANOVA was implemented to the variables corresponding to a two-way factorial design. Experiment 3 was designed with a 2 × 2 factorial structure with blocking effects on the experimental plot. Two-way ANOVA was applied to analyze the response variables. The data from Experiment 4 were analyzed using a simple *t*-test procedure at each time point to see significant differences between control vs. inoculated plants. The numbers of replication for Experiments 1, 2, 3, and 4 were eight, ten, eight, and eight, respectively.

## Results

### Experiment 1: Greenhouse Study Using Multiple Endophyte Strains

**FIGURE 1:**
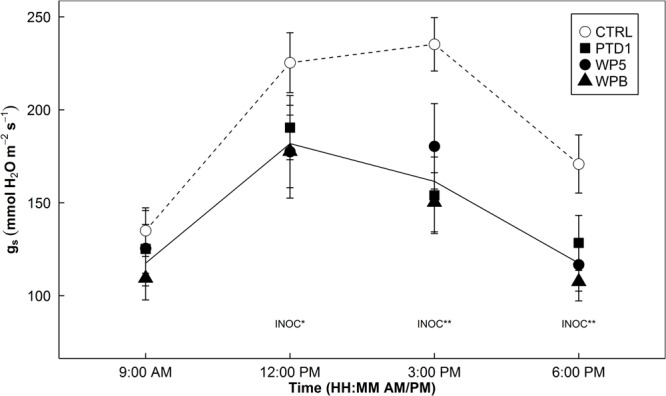
Diurnal patterns of stomatal conductance (*g*_s_) of rice leaves on 163 days after germination in a greenhouse bench experiment (Experiment 1). Open symbols indicate mean *g*_s_ of control groups, whereas closed symbols indicate mean *g*_s_ of single strain-inoculated groups (square/circle/triangle = PTD1/WP5/WPB, individually). Error bars of the means represent ± 1 SE of replicated samples (*n* = 10). Single strain endophyte inoculation effect (INOC) is provided at *P* < 0.05 (^∗^), 0.01 (^∗∗^) levels. Contrast matrix was used to test CTRL vs. INOC (PTD1/WP5/WPB nested) comparison. Dotted and solid lines highlight mean responses of CTRL and INOC plants over time.

Stomatal conductance (*g*_s_) decreased during the daytime in E+ plants with multiple strains of the bacteria (PTD1/WP5/WPB). An average 27% decrease in *g*_s_ by multiple single strain endophytes was found with *P* = 0.124, 0.005, and <0.001 at 12, 3, and 6 pm from Experiment 1 (**Figure 1**).

**FIGURE 2:**
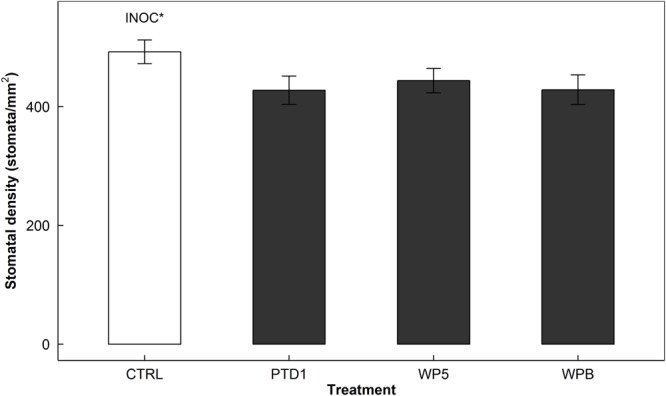
Stomatal density of adaxial sides of rice leaf surfaces on 165 days after germination in a greenhouse bench experiment (Experiment 1). The stomatal imprints were collected from the same leaves that were used in stomatal conductance measurements in **Figure 1**. The bars present mean responses of control (CTRL, open) and the single strain inoculated (PTD1, WP5, WPB from left to right, closed) rice leaves. The error bars indicate ± 1 SE of the means (*n* = 10). Single strain endophyte inoculation effect (INOC) is provided at *P* < 0.05 (^∗^) level. Contrast matrix was used to test CTRL vs. INOC (PTD1/WP5/WPB nested) comparison.

Similar to *g*_s_ response, stomatal density also decreased by 12% (*P* = 0.012) in response to endophyte inoculation (**Figure 2**). That is, compared to 492 stomata/mm^2^ in the control (CTRL), the average stomatal counts were 433 stomata/mm^2^ in the inoculated plants (PTD1/WP5/WPB).

**FIGURE 3:**
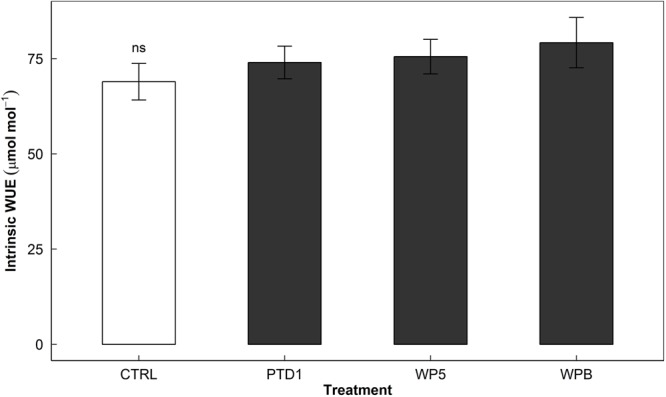
Intrinsic water use efficiency (WUE), expressed as CO_2_ uptake/H_2_O loss in moles, of rice leaves measured on 58/118/153 days after germination in a greenhouse bench experiment (Experiment 1). The bars present aggregated mean responses of control (CTRL, open) and the single strain inoculated (PTD1, WP5, WPB from left to right, closed) rice leaves collected on the 3 days as the *time* effect on the measure were non-significant at *α* = 0.05 level. The error bars indicate ± 1 SE of the means (*n* = 30). Single strain endophyte inoculation effect (INOC) was not significant (ns, *P* = 0.106). Contrast matrix was used to test CTRL vs. INOC (PTD1/WP5/WPB nested) comparison.

Intrinsic WUE, a proxy of short-term WUE, was not significantly changed in E+ plants (*P* = 0.106, **Figure 3**). Overall biomass and total transpiration over time did not respond to endophyte inoculation, suggesting little differences in long-term WUE in Experiment 1 (data not shown).

**FIGURE 4:**
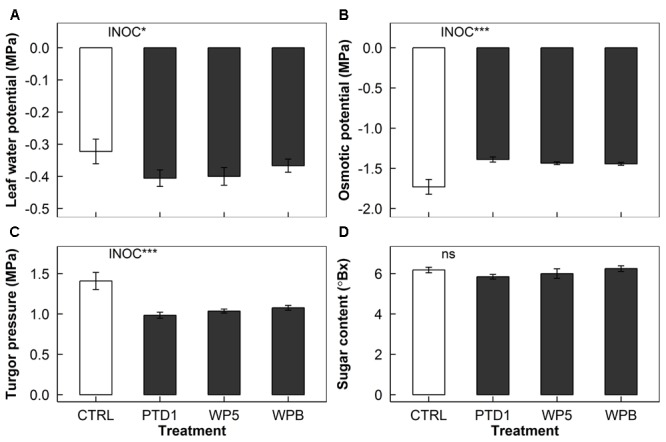
Leaf water potential **(A)**, osmotic potential **(B)**, turgor pressure **(C)**, and soluble sugar content **(D)** of rice leaves on 165 days after germination grown in a greenhouse bench experiment (Experiment 1). The bars present mean responses of control (CTRL, open) and the single strain inoculated (PTD1, WP5, WPB from left to right, closed) rice leaves. The error bars indicate ± 1 SE of the means (*n* = 9). Single strain endophyte inoculation effect (INOC) is provided at *P* < 0.05 (^∗^), 0.001 (^∗∗∗^) level. No significance was found in soluble sugar content (ns). Contrast matrix was used to test CTRL vs. INOC (PTD1/WP5/WPB nested) comparison.

Leaf water potential decreased (21% more negative) in E+ plants regardless of the endophyte strains used (*P* = 0.025, **Figure 4A**). Osmotic potential, on the other hand, was increased (18% less negative) by the endophyte inoculation (*P* < 0.001, **Figure 4B**). This increase in osmotic potential was compensated by the 27% decrease in turgor pressure in the inoculated samples shown in **Figure 4C** (*P* < 0.001). No significant difference was found in soluble sugar content for PTD1/WP5/WPB (**Figure 4D**).

### Experiment 2: CO_2_ Enrichment Study Using a Single Endophyte Strain

**FIGURE 5:**
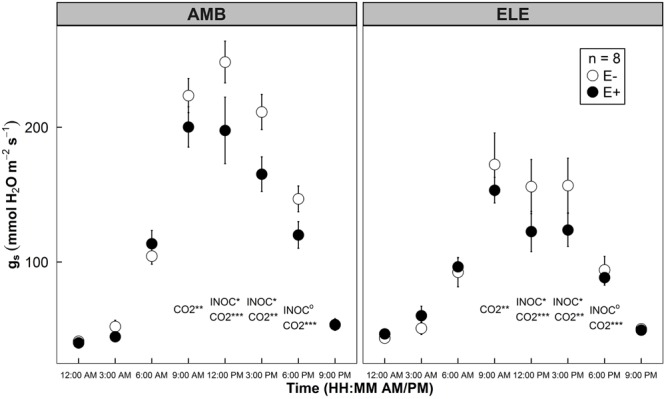
Diurnal patterns of stomatal conductance (*g*_s_) of rice leaves grown under two atmospheric CO_2_ conditions: ambient (AMB, app. 400 ppm on the left panel) and elevated (ELE, app. 800 ppm on the right panel) in a sunlit chamber experiment (Experiment 2). Open symbols indicate mean *g*_s_ of control groups (E–), whereas closed symbols indicate mean *g*_s_ of WP5 inoculated groups (E+). Error bars of the means represent ± 1 SE of replicated samples (*n* = 8). Two-way ANOVA test results are indicated at each time point. CO_2_ treatment effect (CO2) and endophyte inoculation treatment effect (INOC) are provided at *P* < 0.10 (^o^), 0.05 (^∗^), 0.01 (^∗∗^), 0.001 (^∗∗∗^) levels.

Similar to Experiment 1, endophyte inoculation decreased *g*_s_ significantly during the daytime. An average 18% decrease in *g*_s_ by a single strain endophyte was observed with *P* = 0.037, 0.013, and 0.081 at 12, 3, and 6 pm from Experiment 2 (**Figure 5**). No statistical differences were found in the values taken at other time points of the day between AMB and ELE conditions. There were no differences found in measurements made at 9 am between E- and E+ plants (*P* = 0.195 in **Figure 5**). During the peak time of photosynthetic gas exchange activities (12–3 pm), the differences in *g*_s_ became more pronounced, showing 20–21% decreases in E+ plants. High CO_2_ lowered *g*_s_ by 29% across E- and E+ treatments (**Figure 5**).

### Experiment 3: Water Deficit Study Using Endophyte Consortia

**FIGURE 6:**
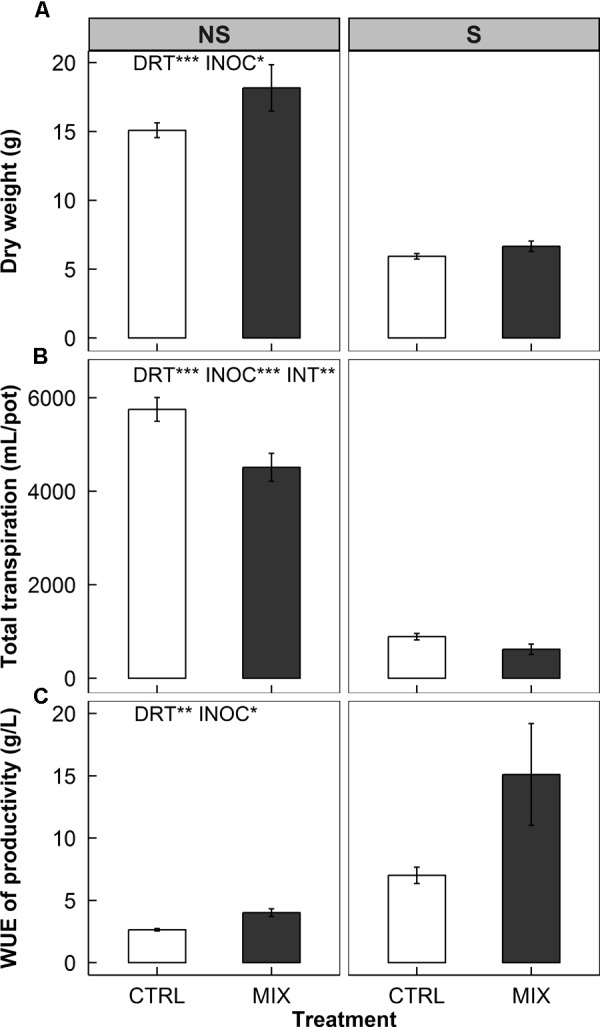
Total biomass (top panels, **A**), total transpiration over time (middle panels, **B**), and water use efficiency (WUE) of productivity (bottom panels, **C**) of rice without (left panels, NS) and with (right panels, S) water deficits at harvest in a greenhouse bench experiment (Experiment 3). Open and closed bars indicate means of mock-inoculated controls (CTRL) and endophyte consortium-inoculated (MIX) plants, provided with error bars as ± 1 SE of the means (*n* = 8). Two-way ANOVA test results of the treatment effects are placed on each panel. Water deficit treatment effect (DRT), endophyte inoculation treatment effect (INOC), and interaction effect (INT = DRT x INOC) are provided at *P* < 0.05 (^∗^), 0.01 (^∗∗^), 0.001 (^∗∗∗^) levels.

We found increases in biomass along with decreases in total transpiration over time and subsequent increases in WUE of productivity of rice plants under both non-stress (NS) and water deficit stress (S) conditions (**Figure 6**).

The water deficit treatment affected all three measures significantly: a decrease in biomass by average 62% (*P* < 0.001, **Figure 6A**), a decrease in total transpiration by an average of 85% (*P* < 0.001, **Figure 6B**), and an increase in WUE of productivity by 221% on average (*P* = 0.002, **Figure 6C**) of the all plants (CTRL and MIX pooled). E+ plants showed an increase in biomass by 16% over E- plants across water deficit treatments (*P* = 0.039, MIX in **Figure 6A**). The endophyte effects on reducing total transpiration were greater under S than NS treatment (*P* = 0.009, the interaction effect – INT – in **Figure 6B**). The magnitudes of the decreases were 30 and 22% in S and NS treatment, respectively (*P* = 0.096 and <0.001).

The endophyte effects on WUE of the NS and S plants combined were significant (84% increase, *P* = 0.047, **Figure 6C**), and this was presumably derived from the decreases in total transpiration (26% decrease, *P* < 0.001, **Figure 6B**) rather than from the increases in biomass (16% increase, *P* = 0.039, **Figure 6A**). The endophyte treatment was more effective in S as the WUE increases were more than twofold compared to NS (116 vs. 52% in **Figure 6C**).

Under NS conditions, the remaining water in MIX was greater than one in CTRL while there was no significant difference in the soil water potential between CTRL and MIX (NS panels in **Supplementary Figure S1**). The water deficit treatment completely dried the remaining water in the buckets and the pots at harvest (S panels in **Supplementary Figure S1**).

### Daily Transpiration Over Daily Light Integrals

**FIGURE 7:**
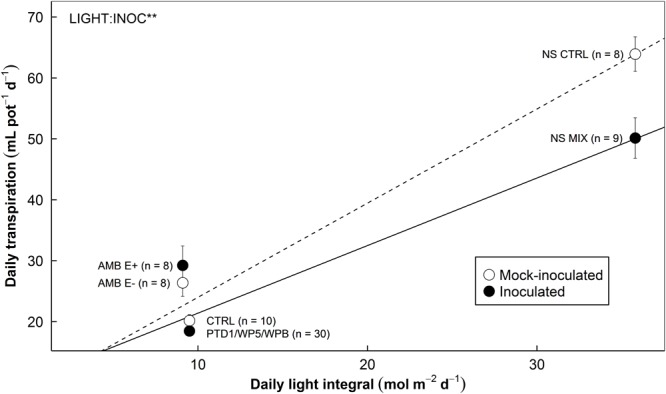
The relationship between daily transpiration by rice plants and daily light integral during growing periods affected by endophyte inoculation in Experiments 1, 2, and 3 (left, middle, and right). Different endophyte and experimental settings were used (refer to **Table 1**). Open symbols (CTRL and E–) stand for the mean responses of mock-inoculated control groups. Closed symbols (PTD1/WP5/WPB, E+, and MIX) stand for the mean responses of endophyte-inoculated treatment groups. Error bars indicate ± 1 SE of the means. The sample sizes are provided in the parentheses. Dotted/solid line show the trends of the responses of control/inoculated groups. Data from elevated CO_2_ in Experiment 2 and water deficit treatment in Experiment 3 are not included in this figure. The relationship is significantly affected by endophyte inoculation (INOC) at *P* < 0.10 level. There is an interaction effect (LIGHT:INOC) at *P* < 0.01 level.

As expected, daily transpiration increased with DLI over the growing periods in both E+ and E- plants (**Figure 7**). The interactions between DLI and endophyte inoculation on daily transpiration were highly significant (*P* = 0.006). This result suggests that reduction in whole-plant transpiration due to endophyte inoculation is more pronounced in high light conditions.

### Experiment 4: *In Vivo* ABA Content Affected by Endophyte Consortia

**FIGURE 8:**
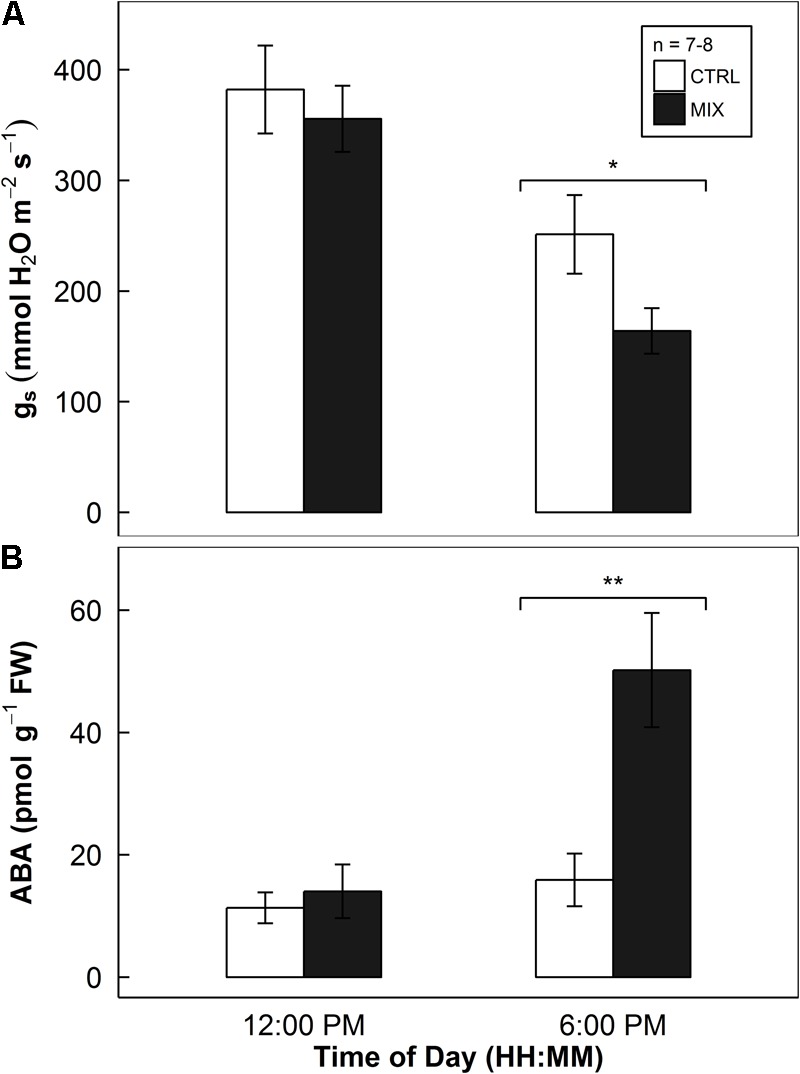
Stomatal conductance of rice leaves at round V7-8 stage (top panel, **A**) and *in vivo* ABA concentrations (bottom panel, **B**) of rice leaves harvested at around R3–R4 stage in a greenhouse sunlit chamber experiment (Experiment 4). Open and closed bars indicate mean responses of mock-inoculated controls (CTRL) and endophyte consortium-inoculated (MIX) plants, respectively, provided with error bars as ± 1 SE of the means (*n* = 7–8). Endophyte inoculation treatment effect (INOC) is provided at *P* < 0.05 (^∗^) and 0.01 (^∗∗^) levels at each time point.

There were no significant differences found in both *g*_s_ and *in vivo* ABA content between E- and E+ rice leaves at noon (**Figure 8**). In the afternoon at 6 pm, the decrease in *g*_s_ by the endophytes was found significant (*P* = 0.043, in **Figure 8A**). Also, the increase in *in vivo* ABA concentrations in E+ rice leaves was found at 6 pm (*P* = 0.006, in **Figure 8B**). The endophyte inoculation caused almost a threefold increase in *in vivo* ABA concentrations in rice leaves.

## Discussion

In the present study, we evaluated the effects of endophyte inoculation on water relations of a rice host. We tested the hypothesis that endophytes alter the host physiology to reduce transpiration of rice through stomatal regulations. A series of experiments conducted in this study illustrated that several changes in water relations took place when a rice plant was inoculated with diazotrophic endophytes. These physiological changes included significantly higher ABA concentrations, lower stomatal conductance, lower stomatal density, lower leaf water potential likely through a reduction in turgor pressure, and eventually an increase in WUE of the whole-plant as these responses accumulate over time and space.

Malinowski and Belesky (2000) reviewed plant physiological mechanisms of drought and mineral stress tolerance offered by cool-season grass endophytes, focusing on the impacts of fungal endophytes. The authors pointed out that some fungal species, even in the same fungal endophyte class, had opposite modes of actions on stomatal reactions and the following plants’ water stress tolerance mechanisms. Similarly, some articles reported increases in *g*_s_ (Bae et al., 2009; Shukla et al., 2012; Gagné-Bourque et al., 2016), while others reported decreases in *g*_s_ (Turner, 1986; Richardson et al., 1993; Elmi and West, 1995), yet highlighting that either action both resulted in the hosts withstanding water deficit conditions.

### Decreases in Stomatal Conductance During Daytime by Endophytes

In Experiments 1, 2, and 4, we observed clear patterns of stomatal behavior with decreases in *g*_s_ in the afternoon (**Figures 1, 5, 8A**). It is evident that stomatal movement of the host plants colonized by the endophytes is heavily dependent on time of day. These experiments performed under different environmental conditions revealed similar patterns of a decrease in *g*_s_ in the afternoon (**Figures 1, 5, 8A**).

Two potential mechanisms may explain this afternoon reduction of stomatal conductance: (1) hormonal influences from the endophytes – endogenous ABA production, or (2) recycling of microbial respiratory CO_2_ in the Calvin cycle of the plants.

The first possibility of altered diurnal stomatal movement is the effects of ABA-production by endophytes. ABA is a key hormone of stomatal control and its diurnal fluctuation is well described by the dual source model (Tallman, 2004). The inoculated plants may have had a higher ABA level due to additional ABA provided by the endophytes, reaching the threshold faster to close stomata. Another related possibility is that endophytes might induce faster circadian clock responses to environmental cues fluctuating in a 24-h cycle (Seo and Mas, 2015). The two-fold increase in WUE by endophytes in Experiment 3 under water deficit conditions would be in line with this respect (**Figure 6C**). This implies the endophytes can cause the host to respond to the environmental changes more sensitively, enabling more efficient use of water as resources.

Stomatal control determines the efficiency of water relations over any other mechanisms, which is governed by ABA concentration (Tardieu and Davies, 1992). All the endophyte strains used in this study produced ABA ranging from 0.404 – 0.831 μg mL^-1^ in an *in vitro* assay from our earlier work (see Table 1 in Khan et al., 2016). Also, we directly showed the increases in *in vivo* ABA concentration along with the decreases in *g*_s_ in the afternoon in Experiment 4 (**Figure 8**). This confirms that the impacts of endophytes on ABA production and resulting decreases in *g*_s_ in rice were significant.

Another piece of evidence supporting ABA production by endophytes is the decreases in stomatal density of the inoculated plants. Together with the stomatal closure during the daytime, it is possible that endophyte-producing ABA affected stomatal development. ABA is also known to involve stomatal development under water deficit conditions (Chater et al., 2014). *In vitro* ABA production capacity of the select nine strains in Experiment 3 was assayed in our previous study, Khan et al. (2016) where the drought tolerance of the inoculated poplar trees was significantly enhanced. This decrease in stomatal density allowed the plants not only to conserve water during the daytime in response to the environmental change, but also may save the metabolic cost to build up guard cells rather than photosynthetic cells. Franks et al. (2015) demonstrated the genetically engineered *Arabidopsis* having less stomata, and therefore so lowered stomatal density, had an advantage over the wild type regarding WUE. Also, Gray et al. (2015) showed that the stomatal density lowered mutant consumed less water over time, displaying higher soil water content than the wild type. Both studies presented the importance of stomatal density as a key control parameter for WUE. Endophyte inoculation can be a novel approach to modify stomatal density as a way to increase WUE.

Our second explanation is the microbial respiration and recycling of CO_2_ molecules by the plants. As *g*_s_ decreased during the afternoon (**Figures 1, 5, 8A**), CO_2_ supply from the atmosphere would drop in E+ plants. However, the increases in WUE (**Figure 6C**) suggest that somehow E+ plants could maintain the rate of photosynthetic CO_2_ assimilation with less CO_2_ through stomata. This implies that other source of CO_2_ supply to the site of carboxylation may possibly contribute to the assimilation process. When carbohydrates are given to endophytes respiring in the intercellular spaces of the leaves, perhaps, this respired CO_2_ is readily available for the Calvin cycle. This endophytic respired carbon would not have to travel through the diffusional pathway of CO_2_, having advantage of the shorter travel distance. Indeed, Bloemen et al. (2013) reported that even CO_2_ respired by root tissues was re-assimilated by the aboveground tissues up to 10–20% in poplar trees. Busch et al. (2013) also demonstrated that up to 24–38% of photorespired and respired CO_2_ were re-assimilated, resulting in an increase of photosynthesis by 8–11% in rice and wheat. These two examples signify how important respired CO_2_ sources from other parts of plants can be in the CO_2_ assimilation process. Although challenging to experimentally prove, our hypothesis – the recycling endophytic respiratory CO_2_ by the hosts’ photosynthesis – holds a possibility.

As opposed to our results with bacterial and yeast endophytes, a meta-analysis from Augé et al. (2014) revealed an average 24% increase in *g*_s_ by mycorrhizae. They conducted the statistical analysis on 400+ individual studies including plants grown under water stressed conditions. Mechanistic differences exist between the two distinguished symbiotic styles. Mycorrhizae aid host plants in absorbing water from the rhizosphere, conferring drought tolerance likely by increasing relative water content of the plants. Conversely, endophytes – from this study – seem to assist host plants in conserving existing water mainly by decreasing *g*_s_.

### Decreases in Leaf Water Potential by Endophytes

Stomatal control comes first in importance before osmotic regulation of water potential in rice plants (Parent et al., 2010). We therefore examined leaf water potential components. The hypothesis behind this was that endophytes would reduce sugar content as they drain different forms of carbohydrates and organic acids from the hosts, leading to decrease in the ability to regulate osmolytes of the plants.

The endophyte inoculation resulted in a reduction in leaf water potential (**Figure 4A**). Interestingly, this reduction was mostly due to a reduction in turgor pressure whereas the osmotic potential increased with inoculation (**Figures 4B,C**). As the endophytes consume carbohydrates produced from the mesophyll cells and are involved in trafficking the transportable form of carbohydrates to the apoplast– mostly sucrose as a basic form (Lemoine et al., 2013) –, osmotic potential of the host cells was likely to be increased (i.e., less negative) (**Figure 4B**). The consumption of other osmolytes (e.g., organic acids) by endophytes can be another possibility of the increase in osmotic potential. As a consequence, the turgor pressure of the cells might have dropped further in part because lower sucrose and organic acid levels in the symplast as they are concurrently consumed and recycled by endophytes. Collectively, with lower *g*_s_ and water potential, the inoculated plants tend to have lower operational cost of water than the control plants.

### Increases in Water Use Efficiency of Hosts

The alterations of stomatal development and diurnal behaviors accompanied with plasticity of cell water relations would offer host plants an advantage of saving water during the daytime, especially under high light and warmer conditions when evapotranspiration demand is high. Although stomata were closed and the CO_2_ supply from the atmosphere to the intercellular, photosynthetic CO_2_ assimilation was not affected by the endophyte inoculation. This advantage is likely to be cumulative over the entire growing period, implying that if there are more sunny days than cloudy and overcast days, the influences will be greater. The differences in our environmental conditions among Experiments 1 through 3 corroborate this point (**Table 1**). We did not find significant increases in biomass and following increases in WUE of productivity in Experiments 1 and 2 (data not shown). Experiment 1 was conducted during mostly winter and early spring featuring the lack of accumulated light intensity and lower air temperature even in the controlled greenhouse environment. Experiment 2 was conducted in the naturally lit growth chambers where the frames of the chambers shaded the plants grown inside and lowered the actual air temperature of the chamber space. On the other hand, the plants grown in the summer season in Experiment 3 showed increases in biomass and long-term WUE (**Figure 6**). Experiment 3 was conducted under full sunlight condition – the average air temperature was approximately 8°C higher and the average DLI was around fourfold higher than the previous two experiments. The reduction in daily water use by endophytes also upholds this point (**Figure 7**). The magnitude of the reduction is positively correlated to DLI.

Another possibility is that a consortium of endophytes would benefit the plants more than a single strain of endophytes. This has to do with the collection of microbiota simulating the natural habitat would help the hosts more by interacting with each other (Bulgarelli et al., 2013). Knoth et al. (2014) also reported a consortium of multi-strain endophytes was more effective on increasing gain of final biomass of poplar clones in a greenhouse experiment. We used single strain endophytes in Experiments 1 and 2 where the decreases in *g*_s_ were observed, but no changes in WUE (**Figure 3**). In Experiment 3, however, where we used a consortium of nine endophyte strains, we found significant increases in long-term WUE (**Figure 6C**).

The decreases in the cumulative total transpiration of the inoculated plants were more significant under the water deficit conditions (**Figure 6B**). Endophytes helping plants under stress is reported in copious articles. Their beneficial effects on the host are thought to be augmented under various stress conditions because the endophytes inside plants signal stress response pathways before the stress is imposed and these microorganisms appear to turn on defense mechanisms of the plants (Pandey et al., 2012).

## Conclusion

We showed that select bacteria and yeast endophytes decreased *g*_s_ suggesting that this stomatal response was the main reason for increases in WUE of the rice plants. The rice plants inoculated with multiple strains of the endophytes all showed the decrease in *g*_s_ and stomatal density. The decrease in *g*_s_ was also observed under a CO_2_ enrichment condition. This stomatal response resulted in reduction of total transpiration of plants over growing periods. The effect size of the reduction was greater when DLI were higher and water supply to plants was limited under water deficit conditions. The reduction in total transpiration was the main reason for increases in long-term WUE in the rice plants. We suggest that the increases in ABA production by endophytes can be a possible mechanism for these stomatal reactions and the resulting whole plant physiological benefits.

## Author Contributions

Conceived idea and designed the experiments: HR and S-HK. Conducted the experiments: HR, NW, and VVE. Analyzed the data: HR and S-HK. Provided the materials and resources: SD and S-HK. Wrote the article: HR, VVE, SD, and S-HK.

## Conflict of Interest Statement

The authors declare that the research was conducted in the absence of any commercial or financial relationships that could be construed as a potential conflict of interest.

## Supplementary Material

FIGURE S1 The remaining water in the buckets (top panels, A) and water potential of the soil in the pots (bottom panels, B) without (left panels, NS) and with (right panels, S) water deficits at harvest. Open and closed bars indicate means of mock-inoculated controls (CTRL) and endophyte consortium-inoculated (MIX) plants, provided with error bars as ± 1 SE of the means (*n* = 8). Two-way ANOVA test results of the treatment effects are placed on each panel. Water deficit treatment effect (DRT), endophyte inoculation treatment effect (INOC), and interaction effect (INT = DRT x INOC) are provided at *P* < 0.01 (^∗∗^) and 0.001 (^∗∗∗^) levels.
