# Supplementary figures and images for: Salicaceae Endophytes Modulate Stomatal Behavior and Increase Water Use Efficiency in Rice
Source: Front Plant Sci. 2018 Mar 2;9:188. doi: 10.3389/fpls.2018.00188 (PMC5840156; doi:10.3389/fpls.2018.00188)

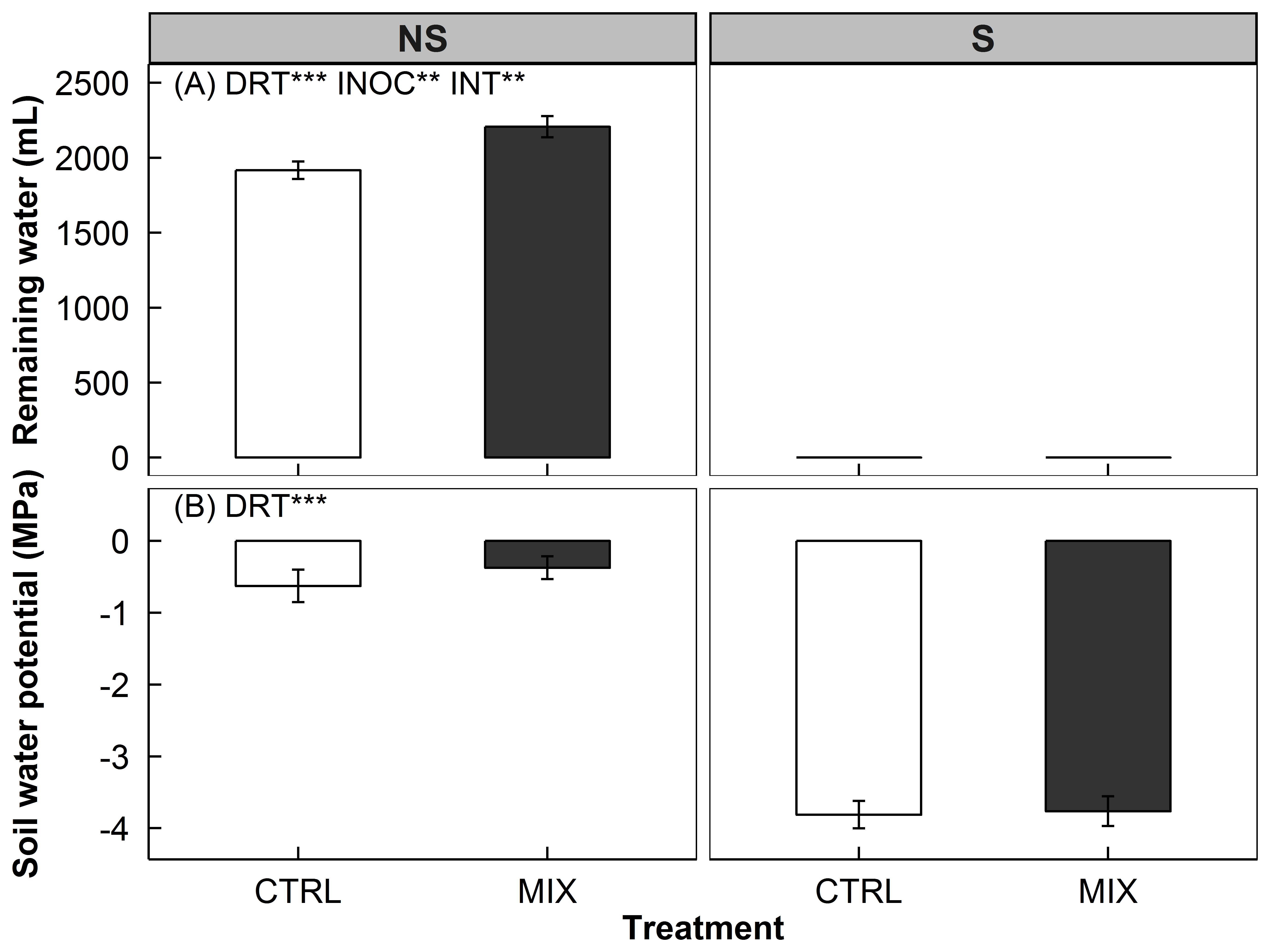

Supplement: FIGURE S1 — The remaining water in the buckets (top panels, A) and water potential of the soil in the pots (bottom panels, B) without (left panels, NS) and with (right panels, S) water deficits at harvest. Open and closed bars indicate means of mock-inoculated controls (CTRL) and endophyte consortium-inoculated (MIX) plants, provided with error bars as ± 1 SE of the means (n = 8). Two-way ANOVA test results of the treatment effects are placed on each panel. Water deficit treatment effect (DRT), endophyte inoculation treatment effect (INOC), and interaction effect (INT = DRT x INOC) are provided at P < 0.01 (∗∗) and 0.001 (∗∗∗) levels. [file Image_1.TIFF]
